# Catheter Ablation Outcomes in Electrical Storm Versus Non–Electrical Storm Ventricular Tachycardia: A Systematic Review and Meta‐Analysis

**DOI:** 10.1002/joa3.70366

**Published:** 2026-07-22

**Authors:** Ahmad Jalil, Fatima Rajab, Xuan Li, Abdulgafar Ibrahim, Arundhati Sawant, Michael Bashir, Varshitha Bandi, Karan Bhatt, Smit Mehta, Bilal Abaid, Arthur Alencar, Mahmoud Gomaa, Muhammad Afzal

**Affiliations:** ^1^ Internal Medicine Department Baptist Hospital‐North Mississippi Oxford Mississippi USA; ^2^ Internal Medicine Department King Edward Medical University Lahore Pakistan; ^3^ Internal Medicine Department Mississippi Baptist Medical Center Jackson Mississippi USA; ^4^ Internal Medicine Department Inspira Health Vineland Vineland New Jersey USA; ^5^ Internal Medicine Department University of Louisville Louisville Kentucky USA; ^6^ Internal Medicine Department Baptist Memorial Hospital Golden Triangle Columbus Mississippi USA; ^7^ Electrophysiology Section The Ohio State University Wexner Medical Center Columbus Ohio USA

## Abstract

**Background:**

Electrical storm (ES) is a life‐threatening manifestation of ventricular tachycardia (VT) associated with highmorbidity and mortality. Catheter ablation is an established therapy for VT, but the impact of ES on ablation outcomes remains uncertain.

**Methods:**

We conducted a systematic review and meta‐analysis in accordance with PRISMA 2020 to compare VT ablation outcomes in patients with and without ES. PubMed, Cochrane Library, and ScienceDirect were searched from inception through January 2026. Eligible studies were comparative cohorts reporting periprocedural or long‐term outcomes. Random‐effects models were used to pool odds ratios (ORs), hazard ratios (HRs), and mean differences (MDs), with heterogeneity assessed using the I^2^ statistic.

**Results:**

Six observational cohort studies including 3531 patients (1183 with ES and 2348 without ES) were included. Acute procedural endpoints, including post‐ablation VT non‐inducibility and residual inducible VT, were similar between groups. Periprocedural or in‐hospital mortality was significantly higher in the ES group (OR 4.71, 95% CI 2.76–8.06), whereas complication rates, procedural duration, radiofrequency ablation time, and need for hemodynamic support did not differ significantly. During follow‐up, ES was associated with higher all‐cause mortality (OR 1.67, 95% CI 1.19–2.35) and greater VT recurrence in both arm‐based (OR 1.45, 95% CI 1.11–1.91) and time‐to‐event analyses (HR 1.35, 95% CI 1.16–1.57).

**Conclusions:**

VT ablation showed comparable acute procedural success and procedural safety in patients with and without ES. However, ES was associated with higher early and long‐term mortality and greater VT recurrence, supporting its role as a marker of adverse prognosis.

## Graphical abstract

Catheter ablation for ventricular tachycardia showed comparable acute procedural success and complication rates in patients with and without electrical storm. However, electrical storm was associated with higher in‐hospital mortality, long‐term mortality, and ventricular tachycardia recurrence, supporting its role as a marker of adverse prognosis.

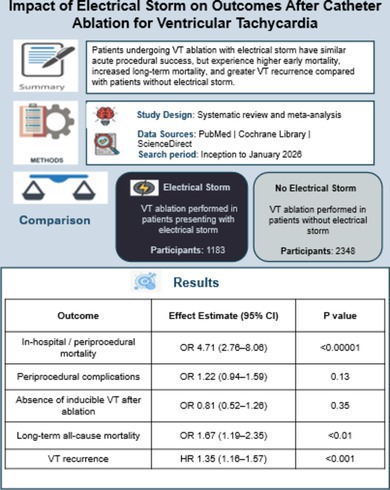

**Abbreviations**
CA: Catheter Ablation
CSD: Cardiac Sympathetic Denervation
ES: Electrical Storm
ICD: Implantable Cardiac Defibrillator
ICM: Ischemic Cardiomyopathy
VT: Ventricular Tachycardia

## 1 Introduction

Ventricular tachycardia (VT) is a severe arrhythmia, and ventricular tachycardia (electrical) storm is characterized by 3 or more episodes of sustained VT within 24 h or incessant VT [1, 2].

Prior studies show that electrical storm (ES) is a life‐threatening condition associated with management challenges and an adverse prognosis compared with isolated VT episodes. ES predominantly occurs in patients with underlying structural heart disease, both ischemic and non‐ischemic etiologies [3].

The occurrence of ES or more episodes of sustained VT affects about 10% of implantable cardioverter defibrillator recipients (ICD). It is a medical emergency, requiring immediate hospitalization in an intensive care unit. About 20% of cases are reversible; approximately 20% of these patients will experience recurrent ES after the initial event, and close to 40% would probably experience recurrent ventricular arrhythmias. The death rate associated with events can be as high as 40% [4]. Additionally, 10%–28% of patients with secondary preventable ICDs can sustain ES associated with an increase in mortality. Studies found arrhythmia caused by monomorphic VT to be more associated with ES compared to polymorphic VT or ventricular fibrillation (VF) [5, 6].

The first line of treatment for ES currently is medical therapy, with the use of anti‐arrhythmic medications. Over the past decade, catheter ablation (CA) has emerged as a highly effective therapeutic strategy for managing ES, particularly in patients in whom medical therapy fails. Research trials have shown that CA is more effective than medical therapy in reducing the recurrence of arrhythmia and improving the outcome for patients with VT with ES [3, 4].

Although CA is an established therapy for VT, the impact of ES on post‐ablation outcomes remains unclear. Prior studies have reported inconsistent findings, with some showing no significant differences between patients with and without ES, while others demonstrate higher rates of VT recurrence and mortality in the ES population [3, 7, 8, 9]. Given these conflicting results and the absence of a comprehensive meta‐analysis on this topic, we conducted a systematic review and meta‐analysis to evaluate acute, periprocedural, and long‐term outcomes of VT ablation in patients with and without ES.

## 2 Methods

### 2.1 Study Design and Registration

This systematic review and meta‐analysis was conducted and reported in accordance with the Preferred Reporting Items for Systematic Reviews and Meta‐Analyses (PRISMA) 2020 statement [10]. Prior to study initiation, the review protocol was prospectively registered in the International Prospective Register of Systematic Reviews (PROSPERO: CRD420251273982).

### 2.2 Literature Search

A comprehensive literature search was performed to identify studies evaluating clinical outcomes of CA for VT, with a focus on comparisons between patients presenting with ES and those undergoing VT ablation without ES. A comprehensive computerized literature search was conducted using the PubMed, Cochrane Library, and ScienceDirect databases from their inception through January 2026. The search strategy combined Medical Subject Headings (MeSH) and free‐text terms using Boolean operators. For PubMed, the following strategy was used (“ventricular tachycardia”[Mesh] OR “ventricular tachycardia”[tiab] OR “VT”[tiab]) AND (“catheter ablation”[Mesh] OR “catheter ablation”[tiab] OR “radiofrequency ablation”[tiab] OR “VT ablation”[tiab]) AND (“electrical storm”[Mesh] OR “electrical storm”[tiab] OR “arrhythmic storm”[tiab] OR “VT storm”[tiab]). For Cochrane Library and ScienceDirect, a similar keyword‐based Boolean strategy was applied (“ventricular tachycardia” OR VT) AND (“catheter ablation” OR “VT ablation” OR “radiofrequency ablation”) AND (“electrical storm” OR “VT storm” OR “ventricular tachycardia storm” OR “arrhythmic storm”). This study was exempt from institutional review board approval and informed consent requirements, as it exclusively analyzed data derived from previously published literature.

### 2.3 Study Selection

Studies were considered eligible for inclusion if they met predefined criteria: (1) human studies involving patients undergoing CA for VT; (2) inclusion of patients presenting with ES as well as a comparator group of patients undergoing VT ablation without ES; and (3) a direct comparison between ES and non‐ES cohorts. Study designs including randomized controlled trials, case–control studies, cohort studies, and other observational studies published in the English language were considered eligible at screening.

Studies were required to report at least one outcome of interest, which were categorized as periprocedural or long‐term outcomes. Periprocedural outcomes included total procedural duration (in minutes), radiofrequency ablation time (in minutes), use of hemodynamic support, in‐hospital or periprocedural mortality, and periprocedural complications. Acute procedural success outcomes included the absence of any inducible VT at the end of the procedure as well as the presence of any residual VT on programmed electrical stimulation at the end of the ablation procedure. Long‐term outcomes included VT recurrence during follow‐up and all‐cause mortality.

**FIGURE 1:**
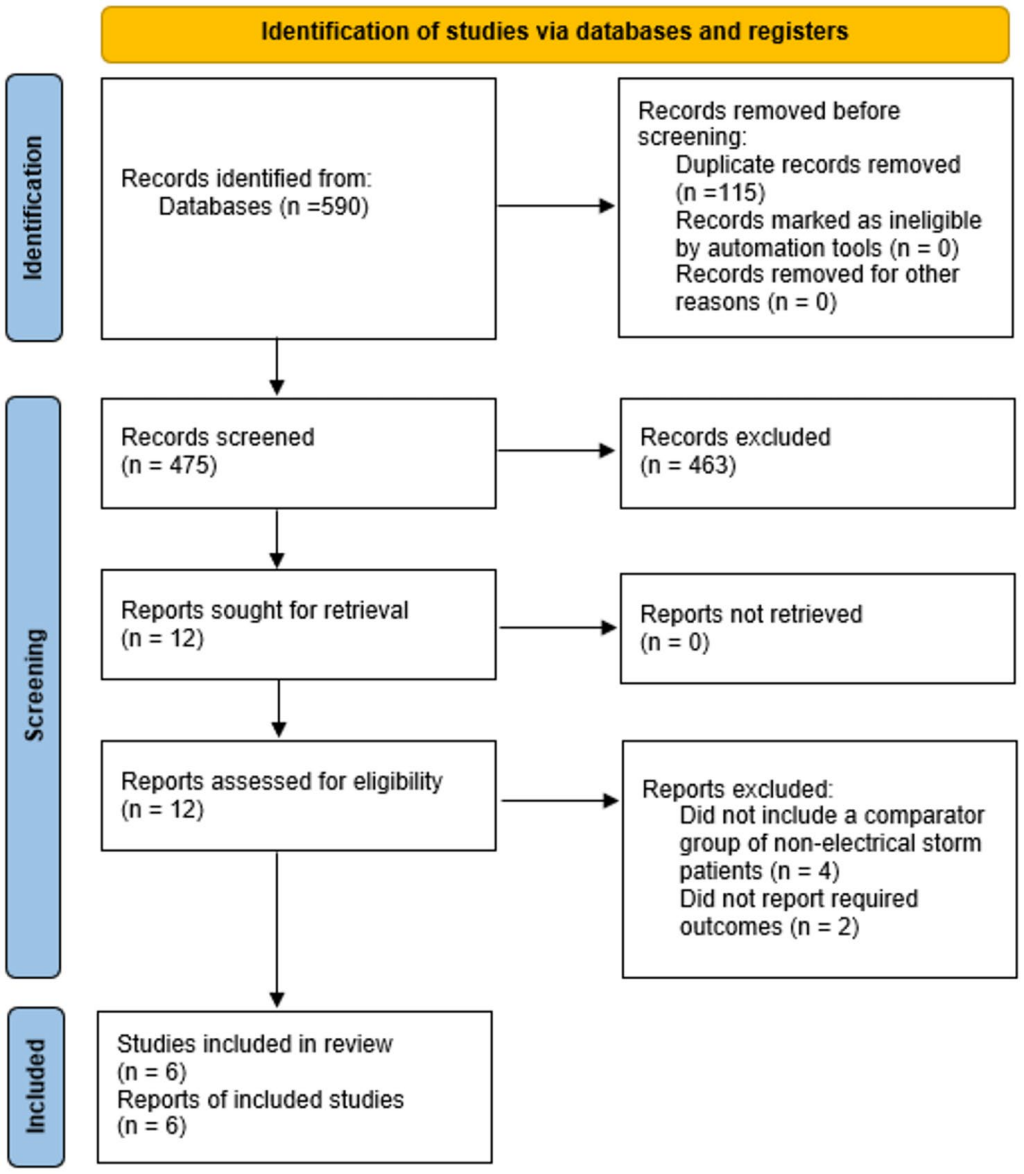
PRISMA Flowchart for included studies.

Studies were excluded if they were in vitro or animal studies; single‐arm studies without a non‐ES comparator group; review articles, systematic reviews, meta‐analyses, editorials; case reports or case series; studies that did not report outcomes of interest; studies in which ES status was not clearly defined; duplicate publications, or studies with overlapping patient populations. A PRISMA flowchart of the search and screening process is presented in (Figure 1).

### 2.4 Data Extraction

Two independent reviewers performed data extraction using a predefined data collection template, with disagreements resolved through discussion. Extracted data included first author and year of publication, study design, sample size, proportion of male participants, mean age, sex distribution, left ventricular ejection fraction (LVEF), proportion of ischemic cardiomyopathy (ICM), history of prior VT ablation, and the prevalence of key comorbid conditions, including hypertension, diabetes mellitus, atrial fibrillation, chronic kidney disease, and ICD use, reported separately for ES and non–ES cohorts when available. All extracted variables were recorded separately for ES and non‐ES groups to ensure direct comparability between cohorts.

Data regarding outcomes of interest was also extracted. Outcomes of interest were categorized as periprocedural and long‐term outcomes. Periprocedural outcomes included total procedural duration, radiofrequency ablation time (in minutes), requirement for hemodynamic support, in‐hospital or periprocedural mortality, and periprocedural complications. Acute procedural success endpoints were extracted according to how they were reported in the included studies, based on programmed electrical stimulation at the end of the ablation procedure, as either VT non‐inducibility (absence of inducible VT) or residual inducible VT (persistence of inducible VT). Long‐term outcomes included VT recurrence and all‐cause mortality during follow‐up.

### 2.5 Quality Assessment

The methodological quality of the studies included was independently assessed by two reviewers. Observational studies were evaluated using the ROBINS‐I tool and traffic plot was constructed using robvis tool, as shown in (Figure S1) [11, 12].

### 2.6 Statistical Analysis

Meta‐analyses were performed using a random‐effects model to account for heterogeneity among studies. For time‐to‐event outcomes, like VT recurrence, multivariable‐adjusted hazard ratios (aHRs) with corresponding 95% confidence intervals (CI) were extracted when available and used for quantitative synthesis, while pooled odds ratios (ORs) were calculated for dichotomous periprocedural outcomes. For the periprocedural/in‐hospital and all‐cause mortality outcomes, pooled odds ratios were derived from study‐level reported event data, as adjusted effect estimates were not consistently available across the included studies. Continuous outcomes, including procedural duration and radiofrequency ablation time, were pooled using mean differences (MDs). All pooled estimates were reported with corresponding 95% CIs. Statistical heterogeneity was assessed using the Higgins I^2^ statistic, with values greater than 50% considered indicative of substantial heterogeneity. All statistical analyses were conducted using Review Manager (RevMan), version 5.4 (The Cochrane Collaboration). A two‐sided *p* value < 0.05 was considered statistically significant.

## 3 Results

The database search resulted in 590 records. After removal of 115 duplicates, 475 unique articles were screened by title and abstract. Following this initial screening, 463 records were excluded. Twelve full‐text articles were assessed for eligibility, of which six studies met the inclusion criteria and were included in the final meta‐analysis.

### 3.1 Study and Patient Characteristics

The characteristics of the included studies are summarized in (Table 1). A total of six studies [3, 7, 8, 9, 13, 14], all of which were observational cohort studies, met the final inclusion criteria and were included in the meta‐analysis. These comprised one large multicenter registry, two multicenter cohorts, and three single‐center retrospective cohorts. Across the six included studies, a total of 3531 patients were analyzed, comprising 1183 patients with ES and 2348 patients without ES undergoing VT ablation. Across studies, patients presenting with ES were generally of similar age compared with non‐ES patients, with mean ages ranging from the early to mid‐60s. Male sex predominated in both groups, exceeding 85% in most cohorts. A history of prior VT ablation was common and tended to be more frequent among ES patients.

**TABLE 1:** Baseline Demographic Characteristics of Patients with Electrical Storm Versus Non–Electrical Storm Undergoing Ventricular Tachycardia Ablation *.

Study (year)	Study design	Total *N*	ES (*n*)	Non‐es (*n*)	Age, es (years)	Age, non‐es (years)	Male, es (%)	Male, non‐es (%)	Prior vt ablation, ES (%)	Prior vt ablation, non‐es (%)
Vergara et al. (2018) [13]	Multicenter registry	1940	677	1263	64.4 ± 12.5	61.3 ± 13.6	89.1	85.9	40.6	38.6
Aldhoon et al. (2017) [3]	Single‐center cohort	328	93	235	64.4 ± 10.6	63.0 ± 12.7	90.3	87.7	NR	NR
Kumar et al. (2016) [14] ICM group	Multicenter cohort	554 (ICM)	186	368	68 ± 10	66 ± 11	88	88	31	31
Mueller et al. (2023) [7]	Single‐center cohort (first VT ablation)	311	108	203	65 ± 14	62 ± 15	86	86	0	0
Certo Pereira et al. (2026) [8]	Single‐center cohort	298	96	202	65 ± 11	64 ± 13.0	90.8	90.1	27.1	16.8
Giraldo et al. (2025) [9]	Single‐center cohort	91	23	77	64	66	67	90	33	12

*Note:* Values are reported as mean ± standard deviation or percentages, as provided in the original studies. Electrical storm was defined according to individual study criteria. For Kumar et al., baseline data were available only for the ischemic cardiomyopathy subgroup and are reported accordingly. NR indicates not reported.

Abbreviations: ES, Electric Storm; VT, Ventricular Tachycardia.

Baseline comorbidities and cardiac characteristics stratified by ES status are summarized in (Table 2). LVEF was consistently lower among ES patients compared with non‐ES patients across most studies, indicating more advanced ventricular dysfunction. ICM was highly prevalent in both groups, particularly in studies restricted to ischemic cohorts, with broadly similar proportions between ES and non‐ES patients. The prevalence of atrial fibrillation, diabetes mellitus, chronic kidney disease, and hypertension was heterogeneous across studies, and not all characteristics were uniformly available.

**TABLE 2:** Baseline Comorbidities and Cardiac Characteristics of Electrical Storm Versus Non–Electrical Storm Patients Undergoing Ventricular Tachycardia Ablation*.

Study (year)	Certo pereira (2026) [8]	Mueller (2023) [7]	Aldhoon et al. (2017) [3]	Vergara et al. (2018) [13]	Kumar et al. (2016) [14]	Giraldo et al. (2025) [9]
AF, ES (%)	34.4	44	NR	33.2	NR	38
AF, Non‐ES (%)	29.2	38	NR	25.3	NR	19
LVEF, ES (%)	30 ± 10	33 ± 14	28.0 ± 9.0	30.4 ± 13.4	27 ± 10	28
LVEF, Non‐ES (%)	36 ± 11	39 ± 15	34.3 ± 12.3	35.2 ± 13.2	31 ± 13	33
ICM, ES (%)	67.7	45	76.3	54.7	100	19
ICM, Non‐ES (%)	66.3	53	70.6	50.8	100	36
DM, ES (%)	28.1	35	29	26.8	25	0
DM, Non‐ES (%)	21.3	23	27.7	18.5	30	11
HTN, ES (%)	77.1	79	64.5	40.2	64	33
HTN, Non‐ES (%)	70.8	78	58.7	45.4	62	30
CKD, ES (%)	51	NR	NR	37.5	41	19
CKD, Non ES (%)	43	NR	NR	26.1	39	23
ICD, ES (%)	99	57	91.4	NR	NR	NR
ICD, Non‐ES (%)	84.2	50	82.1	NR	NR	NR

*Note:* Continuous variables are reported as mean ± standard deviation or median (interquartile range), and categorical variables as percentages. Data was extracted from pre‐matching cohorts when applicable. For Kumar et al., baseline data were available only for the ischemic cardiomyopathy subgroup and are reported accordingly. NR indicates not reported.

Abbreviations: AF, Atrial Fibrillation; CKD, Chronica Kidney Disease; DM, Diabetes Miletus; ES, Electric Storm; HTN, Hypertension; ICD, Implantable Cardiac Defibrillator; ICM, Ischemic Cardiomyopathy; LVEF, Left Ventricular Ejection Fraction.

### 3.2 Acute Procedural Success

**FIGURE 2:**
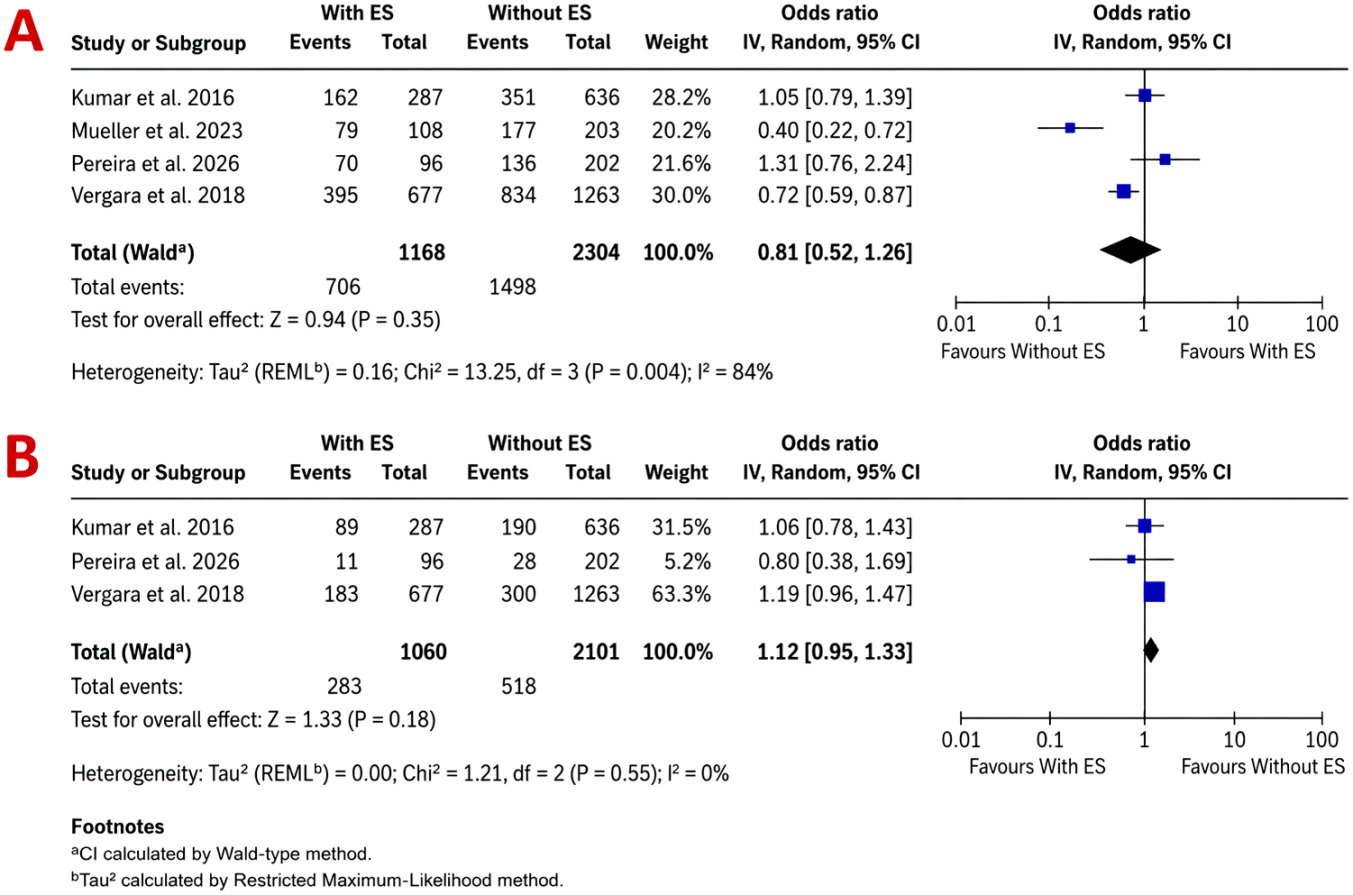
Panel A: Forest plot depicting pooled analysis for absence of inducibility of VT after ablation in patients with and without electrical storm; Panel B: Forest plot depicting pooled analysis for inducibility of VT after ablation in patients with and without electrical storm.

Acute procedural success was assessed using two endpoints: absence of any inducible VT and presence of any residual VT on programmed electrical stimulation at the end of the ablation procedure. For the absence of inducible VT, four observational studies comprising 1168 patients with ES and 2304 patients without ES were included. Pooled analysis using a random‐effects model demonstrated no significant difference between groups (odds ratio [OR] 0.81, 95% CI 0.52–1.26; *p* = 0.35), with substantial heterogeneity across studies (I^2^ = 84%) (Figure 2—Panel A). Similarly, inducibility of VT after ablation was reported in three studies including 1060 ES and 2101 non‐ES patients, with no significant difference observed between groups (OR 1.12, 95% CI 0.95–1.33; *p* = 0.18), and no evidence of between‐study heterogeneity (I^2^ = 0%) (Figure 2—Panel B). Collectively, these findings suggest that the presence of ES does not significantly influence acute procedural success following VT ablation; however, the substantial heterogeneity observed in the non‐inducibility analysis and the observational nature of the included studies warrant cautious interpretation. Additionally, post‐ablation programmed electrical stimulation was not performed in all patients across included studies, and inducibility analyses were therefore limited to tested patients. This may have introduced selection bias, particularly if induction testing was omitted in more unstable or clinically severe patients.

### 3.3 Periprocedural and In‐Hospital Outcomes

**FIGURE 3:**
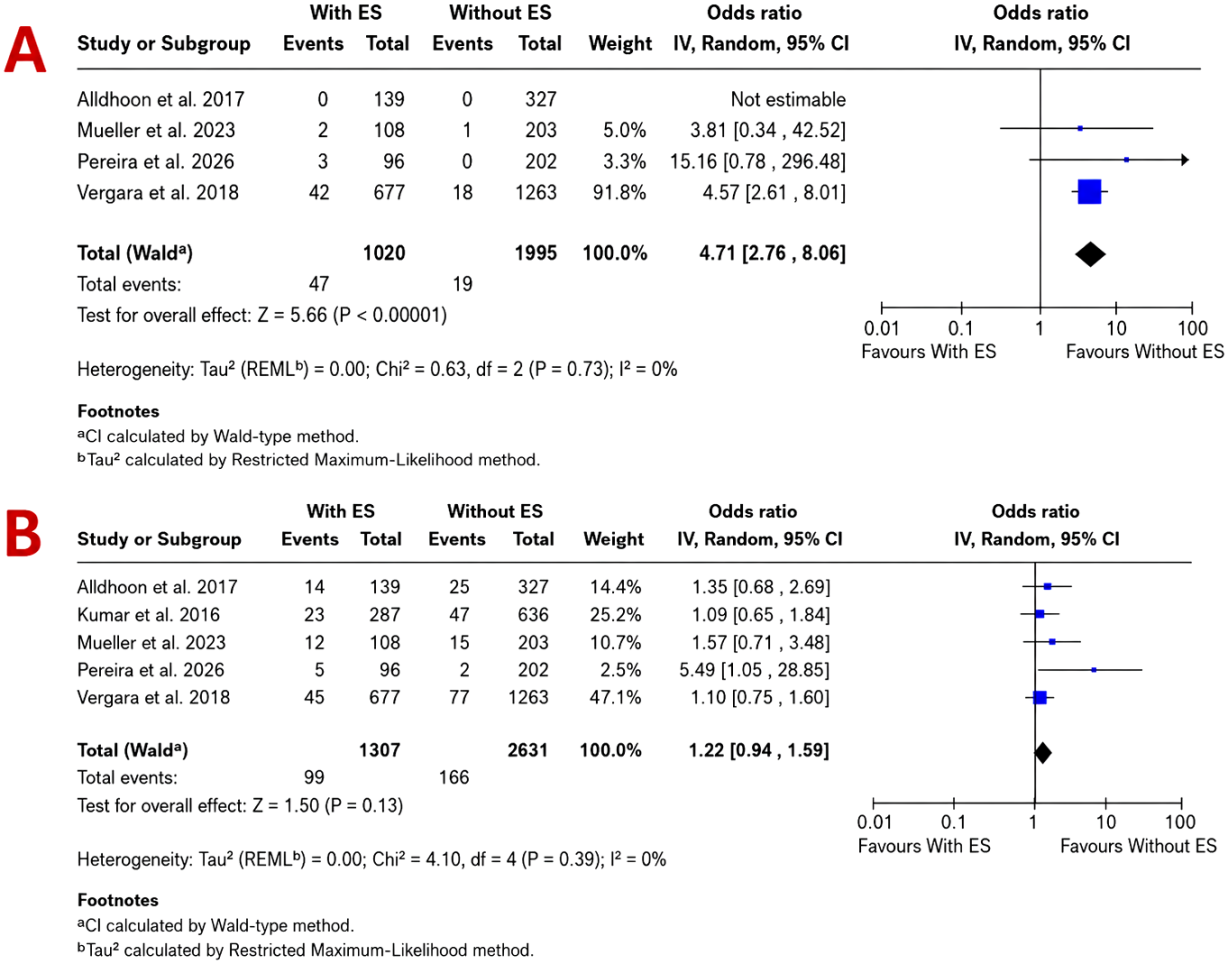
Panel A: Forest plot depicting pooled analysis for periprocedural and in‐hospital mortality outcomes after ablation in patients with and without electrical storm; Panel B: Forest plot depicting pooled analysis for periprocedural and in‐hospital complications after ablation in patients with and without electrical storm.

Periprocedural or in‐hospital mortality and complications were the primary in‐hospital outcomes assessed. Periprocedural or in‐hospital mortality was reported in four observational studies comprising 1020 patients with ES and 1995 patients without ES and was significantly higher among ES patients (odds ratio [OR] 4.71, 95% CI 2.76–8.06; *p* < 0.0001), with no observed heterogeneity (I^2^ = 0%) (Figure 3—Panel A). However, this pooled estimate was largely driven by the study by Vergara et al., which contributed the majority of the statistical weight, and should therefore be interpreted cautiously given the limited number of events in the remaining studies. In contrast, periprocedural or in‐hospital complications, reported in five studies (1307 ES vs. 2631 non‐ES patients), did not differ significantly between groups (OR 1.22, 95% CI 0.94–1.59; *p* = 0.13; I^2^ = 0%) (Figure 3—Panel B).

Procedural aspects were also comparable between groups. The need for periprocedural hemodynamic support, reported in two studies (773 ES vs. 1465 non‐ES patients), was not significantly different (OR 3.03, 95% CI 0.45–20.27; *p* = 0.25), although moderate heterogeneity was observed (I^2^ = 51%). Similarly, no significant differences were observed in radiofrequency ablation time (mean difference 7.07 min, 95% CI −1.28 to 15.42; *p* = 0.10; I^2^ = 81%) or total procedure time (mean difference 9.59 min, 95% CI −6.45 to 25.63; *p* = 0.24; I^2^ = 86%). Overall, these findings indicate that while procedural complexity and complication rates are similar, patients presenting with ES experience substantially higher early mortality, warranting cautious interpretation given heterogeneity in procedural measures and the observational nature of the included studies (Table S1). In addition, because patients with ES generally had worse baseline ventricular function and adjusted effect estimates were not consistently available for this endpoint, the higher in‐hospital/periprocedural mortality observed in the ES group may partly reflect greater baseline disease severity. Notably, the definition and components of procedure‐related complications varied across studies and are summarized in (Table S2).

### 3.4 Long‐Term Outcomes

**FIGURE 4:**
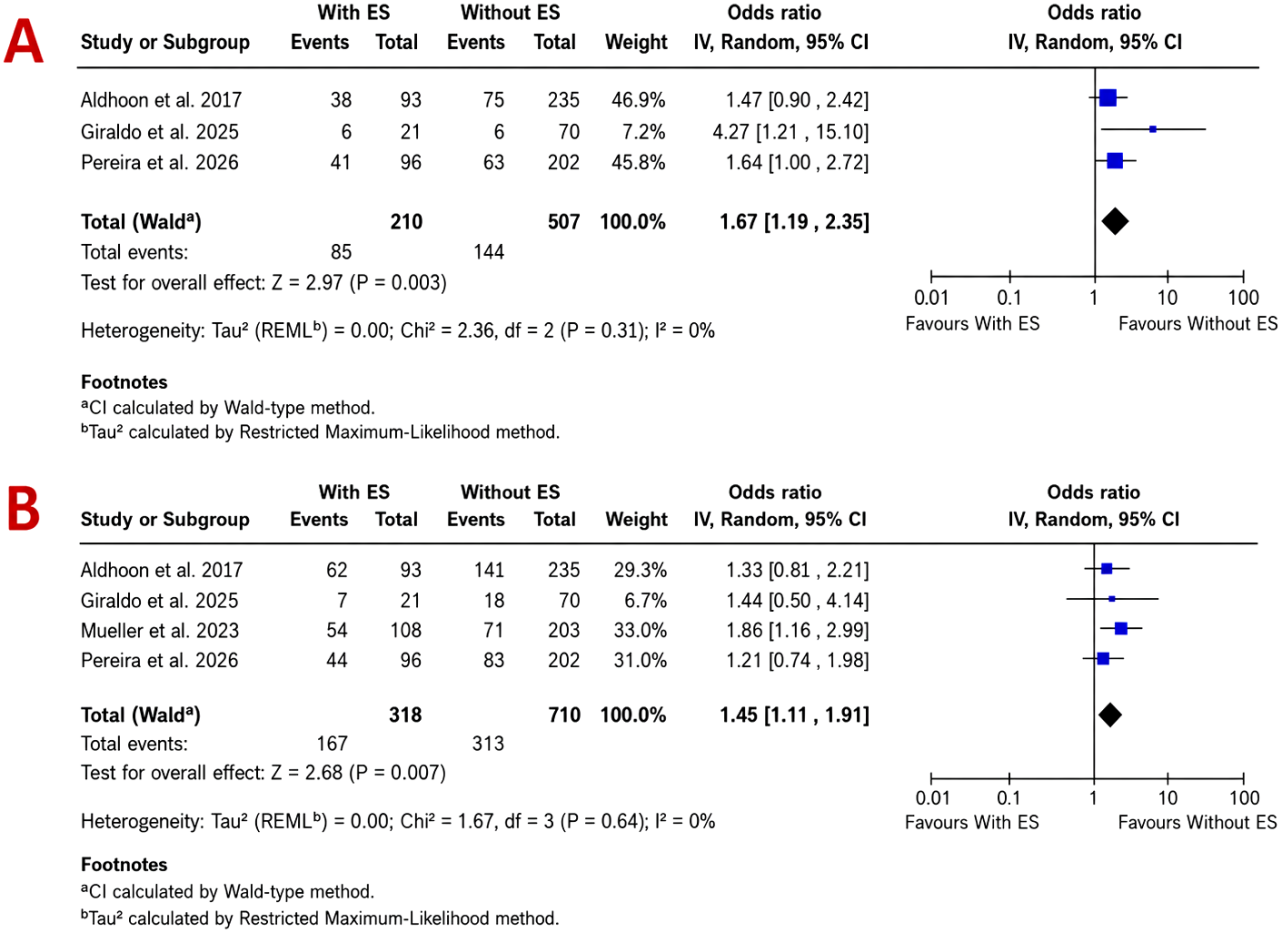
Panel A: Forest plot depicting pooled analysis for all‐cause mortality after ablation in patients with and without electrical storm; Panel B: Forest plot depicting pooled analysis for VT recurrence after ablation in patients with and without electrical storm (using arm‐based data).

Long‐term outcomes included all‐cause mortality and VT recurrence during follow‐up. All‐cause mortality was reported in three observational studies comprising 210 patients with ES and 507 patients without ES. Pooled analysis demonstrated a significantly higher risk of long‐term mortality among ES patients (OR 1.67, 95% CI 1.19–2.35; *p* = 0.003), with no evidence of heterogeneity (I^2^ = 0%) (Figure 4—Panel A). However, because adjusted effect estimates were not consistently available for this endpoint and patients with ES generally had worse baseline cardiac function, this association may partly reflect greater disease severity rather than an independent effect of ES itself. VT recurrence was evaluated using both arm‐based and contrast‐based data. In the arm‐based analysis of four studies (318 ES vs. 710 non‐ES patients), ES was associated with a significantly higher risk of VT recurrence (OR 1.45, 95% CI 1.11–1.91; *p* = 0.007), with consistent findings across studies (I^2^ = 0%) (Figure 4—Panel B). Complementary contrast‐based analyses from four studies reporting time‐to‐event data further supported this association, demonstrating an increased hazard of VT recurrence among ES patients (hazard ratio [HR] 1.35, 95% CI 1.16–1.57; *p* < 0.0001), again with no significant heterogeneity (I^2^ = 0%) (Figure S2).

Collectively, these findings indicate that, despite comparable acute procedural success and largely similar periprocedural complication rates, patients presenting with ES experience significantly worse long‐term outcomes, characterized by both increased mortality and a sustained excess risk of VT recurrence.

## 4 Discussion

### 4.1 Epidemiology and Clinical Context of ES

As mentioned above, ES is a life‐threatening state with instability of cardiac electrics characterized by 3 or more episodes of VT within 24 h, separately by at least 5 min [1, 15]. It has become increasingly common in emergency departments due to increased implantations of ICDs, occurring in about 4%–7% of patients with ICD for primary prevention and 10%–58% of patients with ICD for secondary prevention [16, 17, 18]. The OBSERVational registry on long‐term outcome of ICD patients (OBSERVO‐ICD) showed 4.7% of patients experienced at least one episode of ES during a median follow‐up of 39 months, including 3.9% of patients with ICDs implanted for primary prevention and 10.5% of those implanted for secondary prevention [19]. However, the prevalence of ES in patients without ICD is unclear, as many cases may result in sudden cardiac death outside hospitals [15].

### 4.2 Study Summary and Key Findings

After careful study selection according to the predefined protocol described in the Methods section, this meta‐analysis ultimately included six studies. We provided evidence of outcomes of CA in patients with VT and ES compared to those without ES. Importantly, these associations should not be interpreted as proving an independent adverse effect of ES itself, since patients in the ES group generally had worse baseline ventricular function and more advanced underlying disease, and adjusted effect estimates were not consistently available for all outcomes. According to the results, although all mortality and periprocedural mortality are higher in ES, the periprocedural complications, procedural time, and residual inducible VTs are non‐statistically different between two groups, indicating that CA in ES appears to have similar periprocedural safety and acute endpoints compared with non‐ES. However, these findings should be interpreted with caution with the moderate to serious risk of bias due to confounding we found among our included studies, which may have influenced the observed associations.

Across the studies included, the mean patient age was consistently over 60 years, with no significant difference between the ES and non‐ES groups, except in one study [13]. We also found that the ES group had higher percentages of male patients, chronic kidney disease or elevated creatinine levels, and advanced heart failure, along with older age. One study of them applied matching between groups [7]. Severe cardiac systolic dysfunction, prolonged QRS duration, selected comorbidities such as chronic renal failure (but not diabetes mellitus), advancing age, and male sex are considered risk factors of VT with ES [20, 21, 22]. Other combined chronic diseases in these patients are hypertension, diabetes, stroke, hyperlipidemia, and atrial fibrillation.

### 4.3 Pathophysiology of ES

The detailed mechanisms of ES are not yet clear. However, it is clearer that the development of ES usually needs two factors, arrhythmic substrates and proarrhythmic triggers [15]. As shown in this meta‐analysis, ES is usually associated with other underlying cardiomyopathies and/or structural heart disease. It has been reported that up to 77%–94% of cases are associated with structural heart disease, most commonly cardiomyopathies, including ischemic, non‐ischemic, and arrhythmogenic cardiomyopathy [6]. Other conduction defects (also known as channelopathies) are considered the electrical substrates predisposing to ES, including congenital and acquired long QT syndrome, catecholaminergic polymorphic ventricular tachycardia (CPVT), Brugada syndrome, idiopathic ventricular fibrillation, short QT syndrome, and idiopathic VT [23, 24]. However, there is also a small group of patients with ES who have otherwise normal cardiac conditions but may have molecular abnormalities in their hearts [19, 25]. The proarrhythmic triggers comprise ischemia, electrolyte derangements, heart failure, inflammation, and drug effects [7, 15]. Macro‐reentry is one of the mechanisms of VT with ES, which is caused by slow conduction through ion channels in surviving cells in the scar area leading to monomorphic VT [15, 23, 26]. Other possible mechanisms may include early afterdepolarization and delayed afterdepolarization [27].

### 4.4 Management Strategies for ES

The management for VT and ES is multifactorial, including treatment of the underlying disorder, antiarrhythmic therapy, device therapy, CA, stellate ganglion block, or surgical cardiac sympathetic denervation (CSD) [23, 24]. The first step is to identify and correct any precipitating factors, including underlying heart disease (e.g., acute coronary syndrome and acute heart failure decompensation), electrolyte abnormalities, and drug toxicity [28]. In our study, such adjunctive therapies including antiarrhythmic drugs, mechanical circulatory support, and cardiac sympathetic denervation may have influenced outcomes in patients with ES; however, these variables were not reported consistently enough across the included studies to permit systematic analysis. The ESC guidelines explicitly recommend a stepwise escalation approach starting with ICD reprogramming, sedation, and antiarrhythmic drugs (amiodarone and non‐selective β blockers), progressing to CA, autonomic modulation, and mechanical support as needed [24]. Radiofrequency CA involves the delivery of alternating current at frequencies between 350 and 500 kHz through a catheter‐mounted electrode, resulting in resistive heating and irreversible myocardial tissue injury [29]. In patients with structural heart disease, VT ablation is commonly undertaken using two complementary approaches. Activation mapping defines the full VT activation sequence during ongoing tachycardia, frequently incorporating entrainment mapping to identify the critical isthmus for targeted ablation [26, 30, 31]. Substrate‐based ablation, performed during sinus rhythm, involves detailed characterization of scar architecture and conducting channels, with ablation aimed at eliminating abnormal signals and arrhythmogenic substrate within the scar [26]. Autonomic modulation involves CSD, an established adjunctive therapy for ES, which may be achieved through either percutaneous or surgical approaches, each serving distinct roles in contemporary management [32]. The percutaneous approach, which can be employed as a bridge to definitive therapy, can be achieved through ultrasound‐guided injection of local anesthetics, most commonly bupivacaine, at the stellate ganglion, resulting in acute reduction of ventricular arrhythmia burden. Thoracic epidural anesthesia at the T1–T2 or T2–T3 level represents an additional temporary strategy [32, 33, 34]. Surgical approach involves either open or video‐assisted thoracoscopic approaches and is performed by resecting the lower third to half of the stellate ganglion along with the T2 to T4 or T5 thoracic ganglia, with transection of the nerve of Kuntz when present [32, 35]. Bilateral R1–R4 thoracoscopic sympathectomy has been associated with freedom from recurrent ICD discharges in a substantial proportion of hospital survivors, with estimated overall survival of approximately 73% at 24 months [36]. Overall, catheter ablation is a Class I recommendation for incessant VT or ES caused by monomorphic VT refractory to antiarrhythmic drugs, with the availability of alternative sympathetic denervation strategies in refractory cases. Early ablation during index hospitalization may improve long‐term outcomes compared to conservative management alone [2, 32, 37].

### 4.5 Prognostic Impact and Recurrence in ES

Patients with ES experienced higher long‐term mortality, increased VA recurrence, and a greater burden of ICD therapies, confirming ES as a marker of advanced disease and poorer prognosis. The increased mortality observed in ES likely reflects more severe underlying myocardial pathology, including extensive scar burden, impaired left ventricular function, and progressive structural remodeling [6, 38, 39]. Recurrent ventricular tachyarrhythmias and repeated ICD shocks may further contribute to adverse outcomes by promoting sympathetic activation, myocardial injury, and hemodynamic instability [28, 40]. These mechanisms help explain why ES is associated with both arrhythmic and non‐arrhythmic mortality. Higher rates of VA recurrence and ICD shocks in ES patients highlight the self‐perpetuating nature of this condition. Recurrent arrhythmias facilitate electrical and autonomic remodeling, lowering the threshold for subsequent events and limiting the long‐term effectiveness of antiarrhythmic drug–based strategies [7, 15, 41]. While ICDs are lifesaving, repeated therapies are independently associated with worse prognosis and do not prevent arrhythmia recurrence.

### 4.6 Management Implications and Future Directions

For scar‐related monomorphic VT, ES may indicate a higher arrhythmia burden and, in some patients, a more complex substrate (extensive or heterogeneous scar with multiple re‐entrant circuits) [42]. This substrate complexity may contribute to treatment resistance and high long‐term mortality. In this meta‐analysis, catheter ablation in ES was associated with acute procedural success and periprocedural complication rates comparable to those observed in non‐ES patients, suggesting that ablation remains procedurally achievable in this high‐risk group. However, the higher mortality and VT recurrence observed in ES patients should be interpreted cautiously, as these findings may also reflect greater baseline disease severity and residual confounding. ES should be viewed as an electrophysiological emergency warranting prompt referral to specialized centers and aggressive management. Early and substrate‐directed strategies are critical, while further randomized trials are needed to define the optimal timing of CA in ES.

## 5 Limitations

This meta‐analysis has some important limitations that should be considered when interpreting the findings. First, the number of studies included was limited, with only six eligible publications meeting the predefined inclusion criteria. This reflects the relative scarcity of studies directly comparing outcomes of VT ablation in patients presenting with ES versus those without ES. The small number of studies limits the ability to perform robust quantitative synthesis or subgroup analyses and may reduce the generalizability of the findings. In addition, the available evidence was largely observational, with no randomized controlled trials identified. This is likely related to the acute, life‐threatening nature of ES and the barriers to randomization and prospective controlled study designs. Consequently, the findings are subject to the well‐known limitations of retrospective and observational study designs, including selection bias, confounding, and unmeasured differences between ES and non‐ES populations. Although some studies adjusted for baseline clinical characteristics, residual confounding remains possible especially since the risk of bias due to confounding was moderate to serious among the included studies, which may have influenced the observed associations between ES and worse outcomes. Furthermore, adjusted effect estimates were not consistently reported for important endpoints such as in‐hospital/periprocedural mortality and all‐cause mortality, limiting our ability to determine whether ES was independently associated with these outcomes. By contrast, multivariable‐adjusted hazard ratios were available for VT recurrence in a sufficient number of studies and were preferentially pooled in the time‐to‐event analysis.

There was considerable heterogeneity across studies in terms of patient populations, underlying structural heart disease, ablation strategies, procedural endpoints, and follow‐up duration. Differences in operator experience, mapping techniques, use of substrate‐based versus activation‐guided ablation, and adjunctive therapies (including antiarrhythmic drugs and mechanical circulatory support) may have influenced outcomes and limited direct comparability between studies.

Outcome definitions were not uniform across studies. Acute procedural success, VT recurrence, and complication reporting varied, and follow‐up durations ranged widely, which may have affected the observed long‐term outcomes, particularly VT recurrence and all‐cause mortality. Some studies lacked granular data on cause‐specific mortality or arrhythmic versus non‐arrhythmic deaths. Additionally, adjunctive therapies for ES were not consistently reported across included studies and therefore could not be systematically analyzed, introducing potential residual confounding. Finally, publication bias cannot be excluded, as studies reporting favorable or significant outcomes may be more likely to be published. Although a comprehensive search strategy was employed across multiple databases, unpublished data and non–English‐language studies were not included, which may have further limited the completeness of the evidence base.

Despite these limitations, this review offers a focused synthesis of the limited comparative data available and demonstrates consistent patterns of increased procedural complexity and worse long‐term outcomes among patients undergoing VT ablation in the setting of ES, highlighting the need for larger, prospective studies with standardized definitions and outcomes in this high‐risk population.

## 6 Conclusion

In this systematic review and meta‐analysis, catheter ablation for ventricular tachycardia achieved comparable acute procedural success and periprocedural complication rates in patients presenting with electrical storm and those without electrical storm. However, electrical storm was associated with significantly higher in‐hospital and long‐term mortality, as well as an increased risk of ventricular tachycardia recurrence during follow‐up. These findings indicate that catheter ablation in patients presenting with electrical storm has comparable acute procedural success and periprocedural safety to ablation in those without electrical storm; however, electrical storm remains a marker of advanced myocardial disease and adverse prognosis. Further prospective and randomized studies are needed to define the optimal timing and patient selection for catheter ablation in electrical storm.

## Funding

The authors have nothing to report.

## Ethics Statement

The authors have nothing to report.

## Conflicts of Interest

The authors declare no conflicts of interest.

## Supporting information


**Figure S1:** Risk of Bias assessment for included studies *.
**Table S1:** Pooled analysis of different procedural aspects of VT ablation in patients with and without electrical storm.
**Table S2:** Study‐specific definitions and components of reported procedure‐related complications.
**Figure S2:** Forest plot depicting pooled analysis for VT recurrence after ablation in patients with and without electrical storm (using contrast‐based data).

## Data Availability

The data that support the findings of this study are available on request from the corresponding author. The data are not publicly available due to privacy or ethical restrictions.

## References

[1] C. Çöteli , S. Zekeriyayev , C. Sezer , H. Yorgun , and K. Aytemir , “Long‐Term Outcomes of Catheter Ablation in Ventricular Tachycardia Electrical Storm: A Retrospective Cohort Study,” Clinical Cardiology 48, no. 11 (2025): e70221, 10.1002/clc.70221. 41241775 PMC12619898

[2] K. Huang , R. G. Bennett , T. Campbell , et al., “Early Catheter Ablation Versus Initial Medical Therapy for Ventricular Tachycardia Storm,” Circulation. Arrhythmia and Electrophysiology 15, no. 12 (2022): e011129, 10.1161/CIRCEP.122.011129. 36399370

[3] B. Aldhoon , D. Wichterle , P. Peichl , R. Čihák , and J. Kautzner , “Outcomes of Ventricular Tachycardia Ablation in Patients With Structural Heart Disease: The Impact of Electrical Storm,” PLoS One 12, no. 2 (2017): e0171830, 10.1371/journal.pone.0171830. 28187168 PMC5302378

[4] I. Anagnostopoulos , D. Vrachatis , M. Kousta , et al., “Early Catheter Ablation Versus Conservative‐Only Management in Patients With Electrical Storm. Systematic Review and Meta‐Analysis,” International Journal of Cardiology 438 (2025): 133597, 10.1016/j.ijcard.2025.133597. 40619006

[5] G. N. Kowlgi and Y. M. Cha , “Management of Ventricular Electrical Storm: A Contemporary Appraisal,” Europace 22, no. 12 (2020): 1768–1780, 10.1093/europace/euaa232. 32984880

[6] F. Guerra , M. Shkoza , L. Scappini , M. Flori , and A. Capucci , “Role of Electrical Storm as a Mortality and Morbidity Risk Factor and Its Clinical Predictors: A Meta‐Analysis,” Europace 16, no. 3 (2014): 347–353, 10.1093/europace/eut304. 24096960

[7] J. Mueller , I. Chakarov , P. Halbfass , et al., “Electrical Storm Has Worse Prognosis Compared to Sustained Ventricular Tachycardia After VT Ablation,” Journal of Clinical Medicine 12, no. 7 (2023): 2730, 10.3390/jcm12072730. 37048813 PMC10095385

[8] J. Certo Pereira , R. Barbosa Sousa , D. A. Gomes , et al., “Long‐Term Outcomes of Ventricular Tachycardia Ablation in Patients Presenting With Electrical Storm,” International Journal of Cardiology 444 (2026): 134015, 10.1016/j.ijcard.2025.134015. 41213404

[9] S. Giraldo , J. D. Lopez , R. Isaac , et al., “Ventricular Tachycardia Ablation Outcomes in Electrical Storm,” Europace 27, no. 1 (2025): euaf085.740, 10.1093/europace/euaf085.740.

[10] M. J. Page , J. E. McKenzie , P. M. Bossuyt , et al., “The PRISMA 2020 Statement: An Updated Guideline for Reporting Systematic Reviews,” BMJ (Clinical Research Ed.) 372 (2021): n71, 10.1136/bmj.n71. PMC8005924 33782057

[11] J. A. Sterne , M. A. Hernán , B. C. Reeves , et al., “ROBINS‐I: A Tool for Assessing Risk of Bias in Non‐Randomised Studies of Interventions,” BMJ (Clinical Research Ed.) 355 (2016): i4919, 10.1136/bmj.i4919. PMC5062054 27733354

[12] L. A. McGuinness and J. P. T. Higgins , “Risk‐Of‐Bias VISualization (Robvis): An R Package and Shiny Web App for Visualizing Risk‐Of‐Bias Assessments,” Research Synthesis Methods 12, no. 1 (2021): 55–61, 10.1002/jrsm.1411. 32336025

[13] P. Vergara , R. Tung , M. Vaseghi , et al., “Successful Ventricular Tachycardia Ablation in Patients With Electrical Storm Reduces Recurrences and Improves Survival,” Heart Rhythm 15, no. 1 (2018): 48–55, 10.1016/j.hrthm.2017.08.022. 28843418

[14] S. Kumar , A. Fujii , S. Kapur , et al., “Beyond the Storm: Comparison of Clinical Factors, Arrhythmogenic Substrate, and Catheter Ablation Outcomes in Structural Heart Disease Patients With Versus Those Without a History of Ventricular Tachycardia Storm,” Journal of Cardiovascular Electrophysiology 28, no. 1 (2017): 56–67, 10.1111/jce.13117. 27781325

[15] J. C. Jentzer , P. A. Noseworthy , A. H. Kashou , et al., “Multidisciplinary Critical Care Management of Electrical Storm: JACC State‐Of‐The‐Art Review,” Journal of the American College of Cardiology 81, no. 22 (2023): 2189–2206, 10.1016/j.jacc.2023.03.424. 37257955 PMC10683004

[16] D. Bänsch , D. Böcker , J. Brunn , M. Weber , G. Breithardt , and M. Block , “Clusters of Ventricular Tachycardias Signify Impaired Survival in Patients With Idiopathic Dilated Cardiomyopathy and Implantable Cardioverter Defibrillators,” Journal of the American College of Cardiology 36, no. 2 (2000): 566–573, 10.1016/s0735-1097(00)00726-9. 10933373

[17] A. Verma , F. Kilicaslan , N. F. Marrouche , et al., “Prevalence, Predictors, and Mortality Significance of the Causative Arrhythmia in Patients With Electrical Storm,” Journal of Cardiovascular Electrophysiology 15, no. 11 (2004): 1265–1270, 10.1046/j.1540-8167.2004.04352.x. 15574176

[18] H. W. Sesselberg , A. J. Moss , S. McNitt , et al., “Ventricular Arrhythmia Storms in Postinfarction Patients With Implantable Defibrillators for Primary Prevention Indications: A MADIT‐II Substudy,” Heart Rhythm 4, no. 11 (2007): 1395–1402, 10.1016/j.hrthm.2007.07.013. 17954398

[19] F. Guerra , P. Palmisano , G. Dell'Era , et al., “Implantable Cardioverter‐Defibrillator Programming and Electrical Storm: Results of the OBSERVational Registry on Long‐Term Outcome of ICD Patients (OBSERVO‐ICD),” Heart Rhythm 13, no. 10 (2016): 1987–1992, 10.1016/j.hrthm.2016.06.007. 27291511

[20] A. Arya , M. Haghjoo , M. R. Dehghani , et al., “Prevalence and Predictors of Electrical Storm in Patients With Implantable Cardioverter‐Defibrillator,” American Journal of Cardiology 97, no. 3 (2006): 389–392, 10.1016/j.amjcard.2005.08.058. 16442402

[21] F. Brigadeau , C. Kouakam , D. Klug , et al., “Clinical Predictors and Prognostic Significance of Electrical Storm in Patients With Implantable Cardioverter Defibrillators,” European Heart Journal 27, no. 6 (2006): 700–707, 10.1093/eurheartj/ehi726. 16421175

[22] B. Krzowski , V. Kutyifa , M. Vloka , et al., “Sex‐Related Differences in Ventricular Tachyarrhythmia Events in Patients With Implantable Cardioverter‐Defibrillator and Prior Ventricular Tachyarrhythmias,” JACC Clin Electrophysiol 10, no. 2 (2024): 284–294, 10.1016/j.jacep.2023.09.028. 38032582

[23] S. M. Al‐Khatib , W. G. Stevenson , M. J. Ackerman , et al., “2017 AHA/ACC/HRS Guideline for Management of Patients With Ventricular Arrhythmias and the Prevention of Sudden Cardiac Death: Executive Summary: A Report of the American College of Cardiology/American Heart Association Task Force on Clinical Practice Guidelines and the Heart Rhythm Society,” Circulation 138, no. 13 (2018): e210‐e271, 10.1161/CIR.0000000000000548. 29084733

[24] K. Zeppenfeld , J. Tfelt‐Hansen , M. de Riva , et al., “2022 ESC Guidelines for the Management of Patients With Ventricular Arrhythmias and the Prevention of Sudden Cardiac Death,” European Heart Journal 43, no. 40 (2022): 3997–4126, 10.1093/eurheartj/ehac262. 36017572

[25] T. Noda , T. Kurita , T. Nitta , et al., “Significant Impact of Electrical Storm on Mortality in Patients With Structural Heart Disease and an Implantable Cardiac Defibrillator,” International Journal of Cardiology 255 (2018): 85–91, 10.1016/j.ijcard.2017.11.077. 29425569

[26] S. R. Dukkipati , J. S. Koruth , S. Choudry , M. A. Miller , W. Whang , and V. Y. Reddy , “Catheter Ablation of Ventricular Tachycardia in Structural Heart Disease: Indications, Strategies, and Outcomes‐Part II,” Journal of the American College of Cardiology 70, no. 23 (2017): 2924–2941, 10.1016/j.jacc.2017.10.030. 29216988

[27] F. M. Cauti , P. Rossi , and P. Sommer , “The Sympathetic Nervous System and Ventricular Arrhythmias: An Inseparable Union,” European Heart Journal 42, no. 36 (2021): 3588–3590, 10.1093/eurheartj/ehab168. 33755139

[28] I. Elsokkari , Y. Tsuji , J. L. Sapp , and S. Nattel , “Recent Insights Into Mechanisms and Clinical Approaches to Electrical Storm,” Canadian Journal of Cardiology 38, no. 4 (2022): 439–453, 10.1016/j.cjca.2021.12.015. 34979281

[29] K. Shivkumar , “Catheter Ablation of Ventricular Arrhythmias,” New England Journal of Medicine 380, no. 16 (2019): 1555–1564, 10.1056/NEJMra1615244. 30995375

[30] A. Bhaskaran , R. Tung , W. G. Stevenson , and S. Kumar , “Catheter Ablation of VT in Non‐Ischaemic Cardiomyopathies: Endocardial, Epicardial and Intramural Approaches,” Heart, Lung & Circulation 28, no. 1 (2019): 84–101, 10.1016/j.hlc.2018.10.007. 30385114

[31] S. R. Dukkipati , S. Choudry , J. S. Koruth , M. A. Miller , W. Whang , and V. Y. Reddy , “Catheter Ablation of Ventricular Tachycardia in Structurally Normal Hearts: Indications, Strategies, and Outcomes‐Part I,” Journal of the American College of Cardiology 70, no. 23 (2017): 2909–2923, 10.1016/j.jacc.2017.10.031. 29216987

[32] E. M. Cronin , F. M. Bogun , P. Maury , et al., “2019 HRS/EHRA/APHRS/LAHRS Expert Consensus Statement on Catheter Ablation of Ventricular Arrhythmias,” Heart Rhythm 17, no. 1 (2020): e2–e154, 10.1016/j.hrthm.2019.03.002. 31085023 PMC8453449

[33] Y. Tian , E. D. Wittwer , S. Kapa , et al., “Effective Use of Percutaneous Stellate Ganglion Blockade in Patients With Electrical Storm,” Circulation. Arrhythmia and Electrophysiology 12, no. 9 (2019): e007118, 10.1161/CIRCEP.118.007118. 31514529

[34] T. Bourke , M. Vaseghi , Y. Michowitz , et al., “Neuraxial Modulation for Refractory Ventricular Arrhythmias: Value of Thoracic Epidural Anesthesia and Surgical Left Cardiac Sympathetic Denervation,” Circulation 121, no. 21 (2010): 2255–2262, 10.1161/CIRCULATIONAHA.109.929703. 20479150 PMC2896716

[35] M. Vaseghi , J. Gima , C. Kanaan , et al., “Cardiac Sympathetic Denervation in Patients With Refractory Ventricular Arrhythmias or Electrical Storm: Intermediate and Long‐Term Follow‐Up,” Heart Rhythm 11, no. 3 (2014): 360–366, 10.1016/j.hrthm.2013.11.028. 24291775 PMC4253031

[36] A. C. H. Lee , R. Tung , and M. K. Ferguson , “Thoracoscopic Sympathectomy Decreases Disease Burden in Patients With Medically Refractory Ventricular Arrhythmias,” Interactive Cardiovascular and Thoracic Surgery 34, no. 5 (2022): 783–790, 10.1093/icvts/ivab372. 35015855 PMC9070511

[37] K. Sawalha , F. Kusumoto , M. Al‐Akchar , A. Sadeq , and A. M. Goldsweig , “Catheter Ablation Versus Medical Therapy as First‐Line Treatment for Ventricular Electrical Storm: A Systematic Review and Meta‐Analysis,” Journal of Cardiovascular Electrophysiology 37, no. 2 (2026): 305–312, 10.1111/jce.70222. 41361845

[38] E. Gadula‐Gacek , M. Tajstra , J. Niedziela , Ł. Pyka , and M. Gąsior , “Characteristics and Outcomes in Patients With Electrical Storm,” American Journal of Cardiology 123, no. 10 (2019): 1637–1642, 10.1016/j.amjcard.2019.02.021. 30885418

[39] J. C. Nielsen , Y. J. Lin , M. J. de Oliveira Figueiredo , et al., “European Heart Rhythm Association (EHRA)/Heart Rhythm Society (HRS)/Asia Pacific Heart Rhythm Society (APHRS)/Latin American Heart Rhythm Society (LAHRS) Expert Consensus on Risk Assessment in Cardiac Arrhythmias: Use the Right Tool for the Right Outcome, in the Right Population,” Europace 22, no. 8 (2020): 1147–1148, 10.1093/europace/euaa065. 32538434 PMC7400488

[40] S. Chatzidou , C. Kontogiannis , D. I. Tsilimigras , et al., “Propranolol Versus Metoprolol for Treatment of Electrical Storm in Patients With Implantable Cardioverter‐Defibrillator,” Journal of the American College of Cardiology 71, no. 17 (2018): 1897–1906, 10.1016/j.jacc.2018.02.056. 29699616

[41] J. J. Goldberger , R. Arora , U. Buckley , and K. Shivkumar , “Autonomic Nervous System Dysfunction: JACC Focus Seminar,” Journal of the American College of Cardiology 73, no. 10 (2019): 1189–1206, 10.1016/j.jacc.2018.12.064. 30871703 PMC6958998

[42] J. H. van den Bruck , J. H. Schipper , K. Seuthe , et al., “Preprocedural Substrate Visualization and Image Integration Based on Late Enhancement Computed Tomography for Ventricular Tachycardia Ablation in Non‐Ischemic Cardiomyopathy,” Journal of Clinical Medicine 14, no. 16 (2025): 5801, 10.3390/jcm14165801. 40869628 PMC12387581

