# Supplementary material for: Catheter Ablation Outcomes in Electrical Storm Versus Non–Electrical Storm Ventricular Tachycardia: A Systematic Review and Meta‐Analysis
Source: J Arrhythm. 2026 Jul 22;42(4):e70366. doi: 10.1002/joa3.70366 (PMC13392503; doi:10.1002/joa3.70366)

**Supporting Figure 1: Risk of Bias assessment for included studies ***


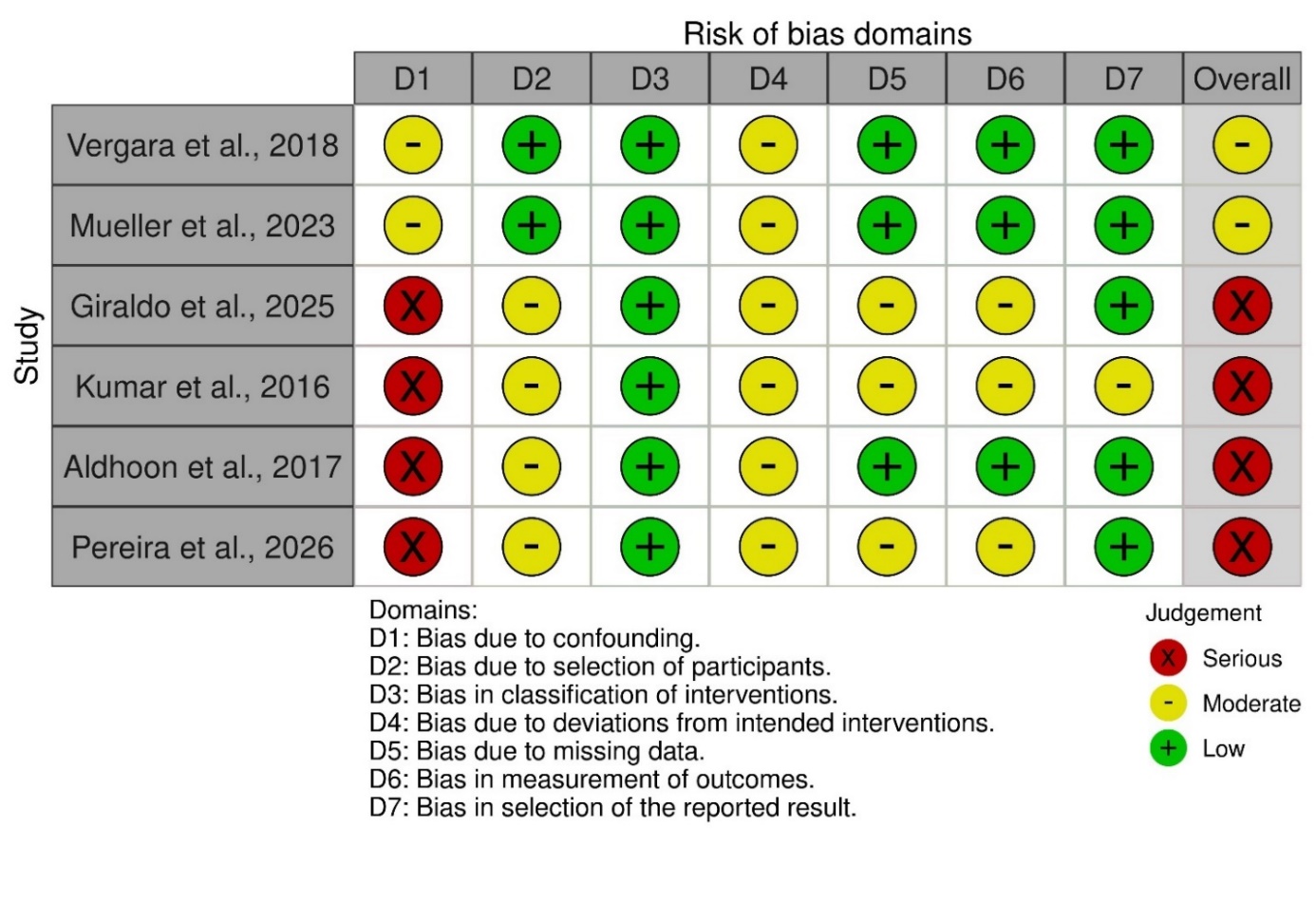


***** Overall, the included studies were judged to have moderate to serious risk of bias, driven primarily by bias due to confounding (D1), reflecting baseline differences and disease severity in patients presenting with electrical storm.

**Supporting Table 1: Pooled analysis of different procedural aspects of VT ablation in patients with and without electrical storm**

| **Outcome** | **Studies (n)** | **Total Patients** | **Effect Estimate** | **95% Confidence Interval** | **P value** |
| --- | --- | --- | --- | --- | --- |
| **In-hospital / periprocedural mortality** | 4 | 3,015 | OR 4.71 | 2.76–8.06 | <0.00001 |
| **Periprocedural / in-hospital complications** | 5 | 3,938 | OR 1.22 | 0.94–1.59 | 0.13 |
| **Periprocedural hemodynamic support** | 2 | 2,238 | OR 3.03 | 0.45–20.27 | 0.25 |
| **Radiofrequency ablation time (minutes)** | 2 | 2,251 | MD 7.07 | −1.28 to 15.42 | 0.10 |
| **Total procedure time (minutes)** | 4 | 3,015 | MD 9.59 | −6.45 to 25.63 | 0.24 |

**Supplementary Table 2:** **Study-specific definitions and components of reported procedure-related complications**

| **Study (Year)** | **Reported Procedure-related Complications** |
| --- | --- |
| **Vergara et al. (2018)**^13^ | Composite procedural complications (individual components not specified). |
| **Aldhoon et al. (2017)**^3^ | *Non-vascular complications:* haemopericardium, strokes, transitory ischemic attacks, thromboembolic event to the left lower limb, cardiac arrests, post-ablation pericarditis, cases of conduction system damage and RV pacing lead dysfunction due to focal ablation adjacent to the lead.  *Vascular complications:* pseudoaneurysms, arteriovenous fistulas, femoral artery injury, and major groin bleeding. |
| **Kumar et al. (2016)**^14^ | Composite procedural complications (individual components not specified). |
| **Mueller et al. (2023)**^7^ | Vascular access-related, third-degree AV block, pneumonia, cardiogenic shock, pneumothorax, stroke. |
| **Certo Pereira et al. (2026)**^8^ | Pericardial tamponade, RV puncture (without tamponade), acute decompensated heart failure, stroke. |
| **Giraldo et al. (2025)**^9^ | Not Available |

**Supporting Figure 2: Forest plot depicting pooled analysis for VT recurrence after ablation in patients with and without electrical storm (using contrast-based data)**


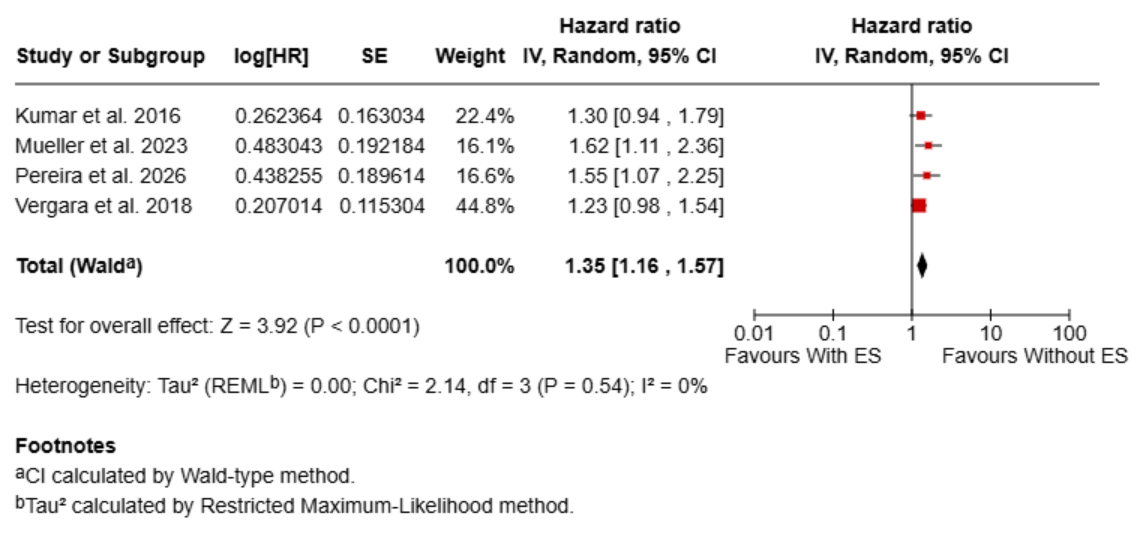

Supplement: Supplementary file 1 — Figure S1: Risk of Bias assessment for included studies *. Table S1: Pooled analysis of different procedural aspects of VT ablation in patients with and without electrical storm. Table S2: Study‐specific definitions and components of reported procedure‐related complications. Figure S2: Forest plot depicting pooled analysis for VT recurrence after ablation in patients with and without electrical storm (using contrast‐based data). [file JOA3-42-e70366-s001.docx]
